# Nutrient, phytochemical, and antinutrient composition of *Citrus maxima* fruit juice and peel extract

**DOI:** 10.1002/fsn3.604

**Published:** 2018-02-20

**Authors:** Peace Nwanneka Ani, Happiness Chiamaka Abel

**Affiliations:** ^1^ Department of Nutrition and Dietetics University of Nigeria Nsukka Nigeria

**Keywords:** *Citrus maxima*, juice, nutrients, peel, phytochemicals

## Abstract

Nutrient, phytochemical, and antinutrient composition of *Citrus maxima* fruit juice and peel extract were determined. The fruit was procured from a garden in Trans‐Ekulu, Enugu East Local Government Area, Enugu State, Nigeria. Mature undamaged *Citrus maxima* fruits were thoroughly washed with distilled water to remove contamination, dirt, and air‐dried. The peel was separated from the pulp. The pulp (100 g) was blended and filtered through a muslin cloth to obtain a clear juice. The peel (50 g) was macerated with 200 ml of ethanol for 20 min. The peel extract was filtered through filter paper. The supernatant was concentrated by rotary evaporation. The peel extract was weighed and stored in a plastic container until needed. Proximate, mineral, vitamins, antinutrient, and phytochemical composition of the juice and peel extract were determined using standard procedures. *Citrus maxima* peel extract contains significantly (*p* < .05) higher crude fiber (2.58%), fat (9.74%), ash (2.49%), and carbohydrate (71.57%) compared with *Citrus maxima* juice. Alkaloid, phenolics, and flavonoids were also significantly (*p* < .05) higher in the peel extract. The mineral composition revealed the order Ca > Na > Ph > Fe > Mg > K in the juice and Ca > Ph > Na > Fe > K > Mg in the peel extract. Vitamin C content of the juice and peel extract were 26.36 mg/100 g and 19.34 mg/100 g, respectively. *Citrus maxima* peel is highly nutritive and rich in phytochemicals, further research is recommended to investigate its therapeutic effect.

## 1 INTRODUCTION

Life in general basically depends on plants. Plant foods are irreplaceable excellent source of nutrients to both man and animals. Many plant species have medicinal value due to many chemical compounds they possess (Joseph & Raj, 2010). The benefits of plants are numerous; the antioxidant components of plants reduce oxidative stress (Agudo et al., 2007). Risk of chronic diseases can be reduced by frequent consumption of fruits and vegetables. Fruit juice is clear or uniformly cloudy unfermented liquid recovered from sound fruits by pressing and other mechanical means (Health & Reineccius, 1986). Citrus is a common term and genus of flowering plant in the family Rutaceae. Citrus is one of the most popular world food crops. Many species are cultivated for their fruit which is eaten fresh or processed into juice. The juice contains a high amount of citric acid giving them their characteristics sharp taste and flavor; they are also good source of vitamin C and flavonoids. The popularity of citrus juice is certainly due to its pleasant and refreshing flavor.

*Citrus maxima* commonly known as shaddock is a tropical plant species which originated from Southeast Asia (Uzun & Yesiloglu, 2012) and are widely cultivated in some regions of West Africa. *Citrus maxima* consists of two parts; the peel and pulp which are easily separated from each other. The pulp is light colored or pink and coarse with large, spindle‐shaped juice sacks. The large shape of the fruit and its colorful peel especially when fully ripe is the major reason it is cultivated as an ornamental tree.

In the past few years, global production of citrus fruit has increased significantly reaching 82 million tons in the year 2009–2010 (USDA: United States Department of Agriculture/Foreign Agricultural Service, 2010). Citrus fruits not only contain large amount of vitamins, minerals, dietary fibers, and pectins but they contain active phytochemicals including phytophenolics (e.g., flavanones, flavones, flavonols, phenolic acids). Citrus peels of various origins have been evaluated for their bioactive compounds. These compounds are widely suggested to exhibit beneficial health effects which include antiatherogenic, anti‐inflammatory, antitumor activity, anticlotting, antimicrobial, and antioxidant activity (Fattouch, Caboni, & Coroneo, 2007; Montanari, Chen, & Widmer, 1998; Samman, Wall, & Cook, 1996). Citrus peels are good sources of molasses, pectin, and limonene and are usually dried with pulp and sold as animal feed (Bocco, Cuvelier, Richard, & Berset, 1998). Citrus fruits are highly consumed as fresh produce and juice, most often the peel is discarded as waste. Production of citrus into juice generates large amount of peel. If not processed further, these wastes produce odor, soil pollution, harborage for insects and can give rise to serious environmental pollution (Shalini & Gupta, 2010).

With the recent gain in popularity of bioactive compounds and concept of functional foods, food products enriched with citrus peels are emerging (Altunkaya, Hedegaard, Brimer, Gökmen, & Skibsted, 2013; Babiker, Sulieman, Elhardallou, & Khalifa, 2013). In this era of food‐based intervention, lack of composition of plant foods and increased wastages poses a major problem to sound nutritional status. To fully utilize the benefit of citrus, documentation of its composition is highly important. The knowledge of chemical composition of C*itrus maxima* juice and peel will enhance their utilization by human, food industries, and pharmaceutical industries, hence the need for this study.

## 2 MATERIALS AND METHODS

### 2.1 Procurement and identification of sample

The fruit was procured from a garden in Trans‐Ekulu, Enugu East Local Government Area, Enugu State, Nigeria. It was identified at the Department of Plant Science and Biotechnology Department, Faculty of Biological Sciences, University of Nigeria; Nsukka as shaddock (*Citrus maxima*).

### 2.2 Preparation of sample

Two mature undamaged *Citrus maxima* fruits were selected, washed thoroughly with distilled water to remove dirt and air‐dried. The fruits were peeled and the peels separated from the pulp. The pulp (100 g) was blended and filtered through a muslin cloth to obtain a clear juice. Fifty grams (50 g) of the peel was macerated with 200 ml of ethanol for 20 min. The peel extract was filtered through filter paper and the residue discarded. The supernatant was concentrated by rotary evaporation. The peel extract was stored in a plastic container until needed.

### 2.3 Chemical analyses

Proximate, vitamin, mineral, antinutrient, and phytochemical compositions of the samples were determined in triplicate.

### 2.4 Proximate analysis

Proximate analysis of the samples was carried out as follows: Moisture content was determined using the hot air method described by Association of Official Analytical Chemist (AOAC (Association of Official Analytical Chemists), 2010). Ash, protein, fat, and crude fiber were determined according to the AOAC (Association of Official Analytical Chemists) (2010) method. Carbohydrate content was determined by difference method. The sum of percentage moisture, ash, protein, fat, and crude fiber was subtracted from 100.

Percentage (%) carbohydrate = 100 – (% moisture + % ash + % protein + % fat + crude fiber).

### 2.5 Vitamin and mineral analyses

Thiamin, pyridoxine, niacin, vitamin E, vitamin C, and folate were determined using the AOAC official methods (Official Methods of Analysis (AOAC), 2000; AOAC (Association of Official Analytical Chemists), 2010). Mineral composition was determined by the procedure described by AOAC (Association of Official Analytical Chemists) (1995). The ash was digested with concentrated hydrochloric acid, transferred to a volumetric flask and made up to 100 ml before the mineral elements (calcium, iron, magnesium, potassium, phosphorus, and sodium) were determined by atomic absorption spectrophotometer (PYE Unicam SP 2900, UK).

### 2.6 Phytochemical and antinutrient analyses

Saponin and oxalate were determined by the Association of Analytical Chemist (AOAC) (2005) method. Flavonoid and alkaloids were determined by the gravimetric procedure of Harborne (1973). Tannins were determined using the Follin‐Dennis spectrophotometric method of Pearson (1976). Phytic acid was determined by the method described by Griffiths and Thomas (1981). Phenolic content was determined using the Folin–Ciocalteu method as described in Dewantoo, Wu, Adam, and Liu (2002).

### 2.7 Statistical analysis

Data collected were statistically analyzed using Statistical Product for Service Solution (SPSS) version 21. They were expressed as means and standard deviation (*SD*).

## 3 RESULTS

The proximate composition of the samples was shown in Table 1. *Citrus maxima* peel extract contains high level of carbohydrate, moderate level of moisture, and fat and low level of protein, crude fiber, and ash. The *Citrus maxima* juice has high moisture, moderate carbohydrate, and low ash, fat, and crude fiber.

**Table 1:** Proximate composition of *Citrus maxima* juice and peel extract (%/100 g)

Proximate composition	Juice	Peel extract
Moisture	79.57 ± 0.06^b^	13.20 ± 0.19^a^
Ash	0.73 ± 0.06^a^	2.49 ± 0.19^b^
Fat	0.83 ± 0.19^a^	9.74 ± 1.01^b^
Protein	1.76 ± 0.19^b^	0.42 ± 0.02^a^
Crude fiber	0.32 ± 0.03^a^	2.58 ± 0.41^b^
Total CHO	16.79 ± 0.23^a^	71.57 ± 0.83^b^

Values are mean ± *SD* of triplicate determinations. Values with different superscripts in the same row are significantly different at *p* < .05.

Vitamin and mineral compositions of *Citrus maxima* juice and peel extract were shown in Table 2. The vitamin content of the juice ranged from 0.55 mg/100 g to 26.36 mg/100 g while that of peel ranged from 1.36 mg/100 g to 279.6 mg/100 g. The vitamins and minerals analyzed were significantly (*p* < .05) higher in *Citrus maxima* peel extract than the juice except for vitamin C which was higher in the juice but this difference was not significant (*p* < .05). The juice contains very low amount of thiamin (0.55 mg/100 g) and folate (0.60 mg/100 g). Mineral content of the juice and peel ranged from 1.30 mg/100 g to132.76 mg/100 g and 5.39 mg/100 g to 515.78 mg/100 g, respectively. Calcium was the most abundant mineral in the peel and juice followed by phosphorus and sodium. The juice contains lower value of magnesium (0.88 mg/100 g) and potassium (1.30 mg/100 g).

**Table 2:** Vitamin and mineral composition of *Citrus maxima* juice and peel extract (mg/100 g)

Vitamin composition	Juice	Peel extract
Thiamin	0.55 ± 0.20^a^	11.20 ± 2.34^b^
Niacin	14.42 ± 1.71^a^	224.16 ± 22.99^b^
Pyridoxine	10.51 ± 4.84^a^	279.63 ± 14.52^b^
Vitamin C	26.36 ± 3.19^a^	19.34 ± 3.75^a^
Vitamin E	2.11 ± 0.08^a^	4.45 ± 0.07^b^
Folate	0.60 ± 0.02^a^	1.36 ± 0.04^b^
Calcium	132.76 ± 2.38^a^	515.78 ± 14.49^b^
Iron	2.17 ± 0.17^a^	9.06 ± 0.79^b^
Magnesium	1.88 ± 0.05^a^	5.39 ± 0.09^b^
Potassium	1.30 ± 0.06^a^	8.75 ± 0.50^b^
Phosphorus	38.96 ± 3.44^a^	366.84 ± 16.47^b^
Sodium	46.12 ± 1.46^a^	274.77 ± 5.07^b^

Values are mean ± *SD* of triplicate determinations. Values with different superscripts in the same row are significantly different at *p* < .05.

Table 3 shows the phytochemical composition of *Citrus maxima* juice and peel extract (mg/100 g). The phytochemical content of the juice ranged from 1.84 mg/100 g to 377.98 mg/100 g while that of juice ranged from 24.51 mg/100 g to 3498 mg/100 g. The peel has significantly (*p* < .05) higher antinutrient content than the juice as shown in Table 4. Phytic acid content of the juice and peel were 86.00 mg/100 g and 444.11 mg/100 g, respectively.

**Table 3:** Phytochemical composition of *Citrus maxima* juice and peel extract (mg/100 g)

Phytochemical composition	Juice	Peel extract
Alkaloid	23.61 ± 2.68^a^	3498.37 ± 917.51^b^
Flavonoid	377.98 ± 20.81^a^	1511.74 ± 35.27^b^
Saponin	1.84 ± 0.18^a^	24.51 ± 0.48^b^
Phenolics	180.86 ± 17.03^a^	1799.04 ± 19.95^b^

Values are mean ± *SD* of triplicate determinations. Values with different superscripts in the same row are significantly different at *p* < .05.

**Table 4:** Antinutrients composition of *Citrus maxima* juice and peel extract (mg/100 g)

Antinutrients composition	Juice	Peel extract
Phytic acid	86.00 ± 5.88^a^	444.11 ± 13.52^b^
Tannin	36.99 ± 17.93^a^	230.04 ± 18.78^b^
Oxalate	63.00 ± 3.12^a^	100.80 ± 8.25^b^

Values are mean ± *SD* of triplicate determinations. Values with different superscripts in the same row are significantly different at *p* < .05.

## 4 DISCUSSION

*Citrus maxima* juice and peel contain appreciable amount of carbohydrate. Carbohydrate was the most abundant macro nutrient in *Citrus maxima* peel. This is in conformity with other studies (Adewole, Adewumi, Jonathan, & Fadaka, 2014; Uraku, 2015; Zahra, Alim‐un‐Nisa, Saeed, Ahmad, & Hina, 2017) which revealed that carbohydrate was the highest occurring macro nutrient in citrus peels. Proximate composition of peels of eight different fruits as evaluated by Feumba, Ashwini, and Ragu (2016) also showed that carbohydrate was the highest macro nutrient in fruit peels. The carbohydrate value of *Citrus maxima* peel in the present study is as high as that of *Citrus vinfera* peel (71.77%) reported by Uraku (2015). *Citrus maxima* peel could be used as a source of carbohydrate especially through incorporation in animal feed production. Carbohydrate is an energy giving nutrient; providing accessible fuel for physical performance and other body functions.

Moisture made up more than three quarter (3/4) of *Citrus maxima juice*. This is expected as citrus is known juicy fruits and is mainly consumed for their ability to quench taste. In addition, the juice was extracted from fresh *Citrus maxima* fruit pulp with no further processing to reduce its liquid content. Moisture content determines the shelf life and the viability of microorganism's growth in a product (Butt, Nasir, Akhtar, & Sharif, 2004). However, processing the juice further by applying pasteurization and canning or bottling can help eliminate activities of microorganisms and increase its shelf life. *Citrus maxima* peel extract contains moderate (13.20%) amount of moisture. This value is comparable to the moisture content of orange peel (10.30%) and *Citrus sinensis* peel (9.78%) reported by Adewole et al. (2014) and Uraku (2015), respectively. The level of fiber in *Citrus maxima* juice was low, this is expected considering the method used in preparing the sample. The fiber content of *Citrus maxima* peel in this study was comparable with the quantity reported in *Vitis vinifera* (4.96%) by Uraku (2015). However, this level was far lower than that obtained with peels of *Citrus sinensis* (13.51%) by Uraku (2015) and orange (14.19%) and pomegranate (17.63%) by Feumba et al. (2016). The differences might be due to the difference in variety of citrus used and method of sample preparation employed. By‐products from fruits have been reported to contain good amount of fiber and functional compounds (Larrauri, 1999). Scientific evidence suggests that increased consumption of fiber (especially dietary fiber) plays a significant role in prevention, reduction, and treatment of chronic diseases and promotion of physiological functions such as lowering blood lipid and glucose control (Figuerola, Hurtado, Estevez, Chiffelle, & Asenjo, 2005). Both the *Citrus maxima* juice and peel extract contain low protein. Protein content of the peel was lower compared with the protein content (9.73% & 16.52%) found in orange peel by Feumba et al. (2016) and Adewole et al. (2014), respectively.

*Citrus maxima* peel contains appreciable amount of fat and ash especially when compared to the value in the juice. The low‐fat content of *Citrus maxima* fruit juice makes it an ideal component in weight reducing diets. The value of fat obtained from the *Citrus maxima* peel is in consonance with the value obtained from *Citrus sinensis* (10.34%) and orange peel (8.70%) by Uraku (2015) and Feumba et al. (2016), respectively. Ash content of a product is a function of the mineral preserved within. The concentration of ash found in *Citrus maxima* peel extract was comparable to the amount found in *Citrus sinensis* (1.59%) and *Vitis vinifera* (4.24%) as reported by Uraku (2015). Ash content of orange peel and pomegranate as reported by Feumba et al. (2016) was also in consonance with the value obtained in this study. The result of proximate analysis of *Citrus maxima* juice in the present study was similar to that reported by Chuku and Akani (2015) for three citrus species (*Citrus sinensis, Citrus limon,* and *Citrus limonia*).

Vitamins are essential organic compounds which are involved in fundamental functions of the body such as growth, maintenance of health and metabolism (Gropper, Smith, & Groff, 2005). Vitamins C and E are antioxidants; they neutralize the effects of free radicals and prevent diseases. *Citrus maxima* juice and peel contain 26.36 mg/100 g and 19.34 mg/100 g of vitamin C. Dipak and Ranajit (2004) reported similar amount of vitamin C in *Citrus maxima* fruit. The peels contain up to 11.20 mg/100 g of thiamin. Deficiency of thiamin in diet can result to overall decrease in carbohydrate metabolism, and its interconnection with amino acid metabolism (via α‐keto acids) has severe consequences, such as a decrease in the formation of acetylcholine for neural function (FAO/WHO, 2001). *Citrus maxima* peel is a good source of niacin and pyridoxine, these vitamins could be harvested from the peel for industrial uses.

The importance of minerals in normal nutrition and metabolism cannot be overemphasized. The mineral composition of fruits largely depends on many factors such as soil type, stage of maturity, variety of cultivars, topography, and other geographical factors. Calcium is a structural component of bones and teeth, it plays role in cellular processes, muscle contraction, blood clotting, and enzyme activation (Gropper et al., 2005). Calcium was the most abundant mineral in *Citrus maxima* juice and peel. This is in line with other studies; Feumba et al. (2016) analyzed the mineral composition of fruit peels and the result showed that orange and pomegranate peels had calcium as the most abundant mineral analyzed. Mineral composition of different varieties of citrus fruits (Okwu & Emenike, 2007) also revealed that calcium was the highest occurring mineral in citrus fruits. Dipak and Ranajit (2004) reported that *Citrus maxima* fruit grown in northern region of Bangladesh had 10 mg/100 g of calcium, which was lower than observed in the present study. The differences could be as a result of factors such as varieties of cultivars and geographical differences.

Sodium is the major positive ion (cation) in extracellular fluid and a key factor in maintaining body fluid. Phosphorus is actively involved in several biologically important compounds such as roles in bone mineralization, energy transfer and storage, nucleic acid formation, cell membrane structure, and acid–base balance (Gropper et al., 2005). The amount of phosphorus and sodium in peel extract of *Citrus maxima* fruit was respectively nine and six times higher than the amount in the juice. Phosphorus content observed in *Citrus maxima* juice (38.96 mg/100 g) was close to that reported in Dipak and Ranajit (2004) but far lower that phosphorus content of other citrus fruits as reported by Okwu and Emenike (2007). Sodium content of *Citrus maxima* juice observed in this study was not in consonance with the value (10 mg/100 g) reported by Dipak and Ranajit (2004).

Iron, magnesium, and potassium are also present in *Citrus maxima* fruit juice and peel extract. Iron is necessary for the proper functioning of the immune system, cognitive development, temperature regulation, energy metabolism, and work performance (Baynes, 2000). Magnesium has a vital role in varying range of biochemical and physiological processes (Schrauzer, 2000). Potassium is recognized for its function in fluid balance and nerve impulse transmission.

Phytochemicals are major bioactive compounds known for their health benefits. The peel extract contains very high amount of alkaloid, phenolics, and flavonoid, saponin was also present in appreciable amount. Phytochemicals were also present in the juice with phenolics and flavonoid being the highest followed by alkaloid and saponin. Studies have shown that plant phenolics not only exist in edible parts of plant but have also been reported in nonedible parts where they exhibit multiple biological effects. Phenolics in fruits are important in reducing risk of heart disease by controlling cholesterol accumulation (Meyer, Yi, Pearson, Waterhouse, & Frankel, 1997). Phenolics in functional foods have antioxidant effects, and its mechanism involves free radical scavenging and metal chelating activities. Wang et al. (2014) reported that peels of citrus fruits possess high amount of flavonoid compared with other edible parts of the fruit. Citrus flavonoids have many beneficial properties which include anticancer, antiviral, and anti‐inflammatory activities reduced capillary fragility and restrict human platelet aggregation (Benavente‐Garcia, Castillo, Francisco, Ana‐Ortuno, & Delrio, 1997; Huet, 1982). Citrus ability to reduce blood glucose and lipid profile level in human has been accredited to two flavonoids; naringin and neohesperidin (Zhang et al., 2012). Saponin has been reported to reduce uptake of certain nutrients such as glucose and cholesterol at the gut through intraluminal physicochemical interactions. They have hypoglycemic and hypocholesterolemic effects (Price, Johnson, & Ferewick, 1987). Biological activities of alkaloids are well documented; including antitumor, diuretic, antiviral, antihypertensive, antidepressant, antimicrobial, and anti‐inflammatory (Aberoumand, 2012).

Antinutritional compounds are relatively high in *Citrus maxima* peel extract compared to the juice with phytic acid being the highest among the three antinutrients analyzed. Antinutrients are compounds or substances which act to reduce nutrient intake, digestion, absorption, and utilization and may produce other adverse effects (Aberoumand, 2012). Tannin binds to and precipitates protein and various other organic compounds including amino acids and alkaloids. Phytic acid binds essential minerals such as calcium, magnesium, iron, and zinc to form insoluble phytates (Agte, Tarwadi, & Chiplonkar, 1999). Oxalate in plant is known to form complex with essential trace metals thereby making them unavailable for physiological and biochemical roles (Ladeji, Akin, & Umaru, 2004). It is important to note that being an antinutrient is not an intrinsic characteristic of a compound but depends upon the digestive process of the ingesting animal. The beneficial antinutritional properties also depend on the amount ingested. In addition, subjecting antinutrients to heat treatment renders them inactive (Iyayi, Kluth, & Rodehutscord, 2008).

## 5 CONCLUSION

*Citrus maxima* juice and peel are good sources of important nutrients and phytochemicals. Incorporation of *Citrus maxima* peel in food products for human consumption is an important way to not only boast the flavor and acceptability of the product but also increase its phytochemical and nutrient densities. The food products can help reduce the risk of chronic diseases and promote physiological functions. This study has shown that *Citrus maxima* juice and peel are quite nutritive and have medicinal properties.

## CONFLICT OF INTEREST

None declared.

## ETHICAL REVIEW

This study does not involve any human or animal testing.
